# Gender Differences in the Path to Medical School Deanship

**DOI:** 10.1001/jamanetworkopen.2024.20570

**Published:** 2024-07-05

**Authors:** Maya S. Iyer, Carol Bradford, Amy S. Gottlieb, David B. Kling, Reshma Jagsi, Christina Mangurian, Lilly Marks, Carolyn C. Meltzer, Barbara Overholser, Julie K. Silver, David P. Way, Nancy D. Spector

**Affiliations:** 1 Department of Pediatrics, The Ohio State University College of Medicine, Columbus; 2 Division of Emergency Medicine, Nationwide Children’s Hospital, Columbus, Ohio; 3 Department of Otolaryngology, The Ohio State University College of Medicine, Columbus; 4 Department of Obstetrics and Gynecology, Keck School of Medicine, University of Southern California, Los Angeles; 5 Department of Internal Medicine, Keck School of Medicine, University of Southern California, Los Angeles; 6 Department of Radiation Oncology, Emory University School of Medicine, Atlanta, Georgia; 7 Department of Psychiatric and Behavioral Sciences, University of California, San Francisco School of Medicine, San Francisco; 8 Department of Epidemiology and Biostatistics, University of California, San Francisco School of Medicine, San Francisco; 9 University of Colorado and Anschutz Medical Campus, Aurora, Colorado; 10 Department of Radiology, Keck School of Medicine, University of Southern California, Los Angeles; 11 Executive Leadership in Academic Medicine (ELAM) Program, Philadelphia, Pennsylvania; 12 Department of Physical Medicine and Rehabilitation, Harvard Medical School, Boston, Massachusetts; 13 Department of Pediatrics, Drexel University College of Medicine, Philadelphia, Pennsylvania

## Abstract

**Importance:**

Women account for only 28% of current US medical school deans. Studying the differences between women and men in their preparation to becoming deans might help to explain this discrepancy.

**Objective:**

To identify differences in the leadership development experiences between women and men in their ascent to the medical school deanship.

**Design, Setting, and Participants:**

In this qualitative study, volunteers from the roster of the Association of American Medical Colleges Council of Deans were solicited and interviewed from June 15 to November 9, 2023. Women deans were recruited first, then men who had been appointed to their deanships at a similar time to their women counterparts were recruited. Deans were interviewed on topics related to number of applications for deanships, prior leadership roles, leadership development, personal factors, and career trajectories. Interviews were coded, and themes were extracted through conventional content analysis.

**Main Outcome and Measures:**

Career and leadership development experiences were elicited using a semistructured interview guide.

**Results:**

We interviewed 17 women and 17 men deans, representing 25.8% (34 of 132) of the total population of US medical school deans. Most deans (23 [67.6%]) practiced a medicine-based specialty or subspecialty. No statistically significant differences were found between women and men with regard to years to attain deanship (mean [SD], 2.7 [3.4] vs 3.7 [3.7] years), years as a dean (mean [SD], 5.7 [5.2] vs 6.0 [5.0] years), highest salary during career (mean [SD], $525 769 [$199 936] vs $416 923 [$195 848]), or medical school rankings (mean [SD], 315.5 [394.5] vs 480.5 [448.9]). Their reports indicated substantive gender differences in their paths to becoming a dean. Compared with men, women deans reported having to work harder to advance, while receiving less support and opportunities for leadership positions by their own institutions. Subsequently, women sought leadership development from external programs. Women deans also experienced gender bias when working with search firms.

**Conclusions and Relevance:**

This qualitative study of US medical school deans found that compared with men, women needed to be more proactive, had to participate in external leadership development programs, and had to confront biases during the search process. For rising women leaders, this lack of support had consequences, such as burnout and attrition, potentially affecting the makeup of future generations of medical school deans. Institutional initiatives centering on leadership development of women is needed to mitigate the gender biases and barriers faced by aspiring women leaders.

## Key Points

**Question:**

Are the paths toward deanships of US medical schools different between women and men?

**Findings:**

This qualitative study of 34 US medical school deans (representing 25.8% of the total population of US medical school deans) found that compared with men, women needed to work harder to overcome a lack of organizational support and other biases they faced during their leadership ascent. Women were similar to men in the number of attempts and years taken to attain deanships.

**Meaning:**

This study suggests that institutions should provide midcareer women equivalent levels of organizational support, sponsorship, and cultivation of their leadership potential that their male counterparts appear to receive more routinely on their paths to deanships.

## Teaser

This qualitative study examines factors associated with differences in the leadership development experiences, including organizational support, sponsorship, and cultivation of leadership potential, between women and men in their ascent to medical school deanship.

## Introduction

Over the past 30 years, the percentage of women medical school deans in the US has steadily increased, but at 28% as of 2023, it still remains far below the percentage of men in these positions.^1,2^ Historically, medical school deans have been White men in their 50s, former department chairs, whose primary specialty was internal medicine.^3,4^ Conversely, prior to pursuing a medical school deanship, women physicians have been particularly underrepresented in positions that involve complete leadership oversight of departments, centers and units responsible for research, clinical delivery, corporate strategy, or policymaking. Women are overrepresented in leadership roles involving education; diversity, equity, and inclusion; mentoring; and institutional public image.^5,6,7^ The current proportion of women in medical college staff leadership roles remains consistent with historic trends: 18% of department chairs, 47% of associate deans, and 52% of assistant deans,^6^ raising the question whether there is a difference in the path to deanship.

Among medical school deans, gender differences in leadership oversight are apparent. Women deans serve at schools with lower National Institutes of Health (NIH) research award rankings and have narrower responsibilities in their role as dean.^8^ Women deans also more commonly oversee just the medical school, compared with men deans who more commonly additionally oversee faculty practice plans, other health professional schools, or hospital and health systems.^8,9^ Women also earn less than their peers in the same decanal administrative roles.^10^ Despite these systemic barriers, there are no differences between women and men deans with regard to the mean number of NIH grants or mean total NIH funding.^11^

Studies have repeatedly highlighted individual and systemic barriers that women physicians, including women physicians from racial and ethnic minority groups, have faced in leadership ascent, including hidden curricula, toxic workplace environments, sexual harassment, juggling caregiving with professional responsibilities, and navigating embedded hierarchical organizational structures.^12,13,14,15,16^ Data from the corporate world suggests that “for each woman who achieves a leadership position in the first decade of her career, there are 1.8 men doing the same…for members who first reach a leadership position during their career’s second decade, the ratio is 2.3 men for every woman.”^17^ A Hewlett Packard internal report showed that women working at the company applied for promotion only when they believed they met 100% of the job requirements, whereas men applied when they thought they had met 60%.^18^

If we are to recognize that medical school deans not only represent and embody the mission of their institutions but also influence the future direction of medicine as a profession, diversification of the deanships is needed. Given the current underrepresentation of women in medical school dean positions, we sought to understand whether there were differences in the path to deanship between women and men with regard to number of years and attempts to attain the deanship and career and leadership development.

## Methods

The institutional review boards of Nationwide Children’s Hospital and Drexel University College of Medicine approved this study. We obtained verbal consent from participants. We followed the Consolidated Criteria for Reporting Qualitative Research (COREQ) reporting guideline.

### Study Design

We conducted a qualitative descriptive study comparing women and men medical school deans. The goal of qualitative description is to stay true to the participants’ perceptions and experiences, while presenting the findings in a straightforward manner.^19,20,21^ We selected this method given that the study aim was to provide a description of women and men deans’ professional and personal journeys to deanship and identify gender differences in their experiences.

### Sampling and Recruitment

We obtained permission from the Association of American Medical Colleges (AAMC) Council of Deans to access their roster of 170 US medical school deans as of 2023. This roster included name, title, organization, and postal address. We obtained email addresses and year of deanship appointments from website searches. We excluded deans of non-US medical schools, interim deans, nondecanal positions (eg, vice, senior associate, associate, or assistant deans), and those retiring prior to our data collection period (Figure). We contacted 100% (N = 36) of US medical school women deans through emails, describing the study and inviting participation. We randomly selected men deans whose appointments started around the same time as the women who agreed to participate and then contacted them via email. We continued recruitment until we had 2 equal groups of women and men interviewees.

**Figure.**
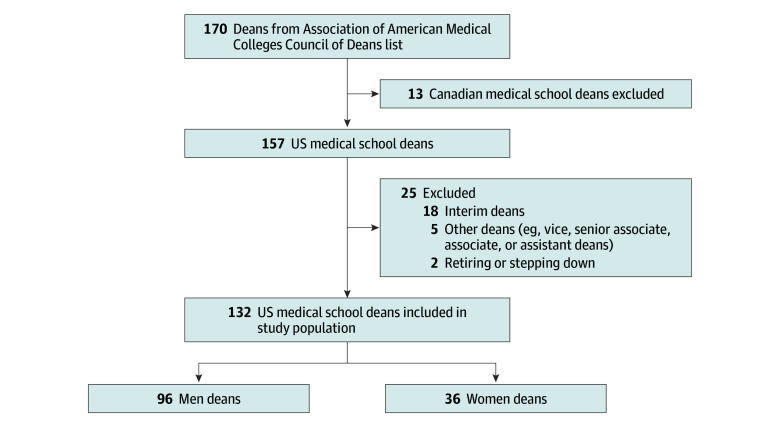
Population of US Medical School Deans Based on 2023 Association of American Medical Colleges Council of Deans List

We developed a 9-item semistructured interview guide through iterative review before piloting the interview with dean’s cabinet leaders (associate and assistant deans) who had similar career aspirations and trajectories as the deans but were not part of the study population (eAppendix in Supplement 1). This pilot interview served as 1 method of data verification, methodological coherence, wherein the questions match the research method, which in turn matches the data and the analytic procedures.^22^ The interview guide also asked for demographic information, including self-identified gender and race and ethnicity (see the eAppendix in Supplement 1 for items). We assessed race and ethnicity to understand if possible intersectional identities played a role in paths to deanships. A single interviewer (M.S.I.) conducted telephone interviews with participants between June 15 and November 9, 2023. Interviews were securely recorded, deidentified, and transcribed (TranscriptionWing; 2024).

### Statistical Analysis

Although the study focus was on the qualitative interviews, we used statistical analyses to confirm that our sample of women and men deans were demographically similar on several key variables, including number of years to attain deanship, number of years as dean, deanships applied for, deanships nominated for, unsolicited applications offered, times as a finalist, and highest salary during their careers. We summarized demographics using descriptive statistics and used independent *t* tests and effect sizes to compare genders.^23^ We controlled for type I error rates using a Bonferroni correction to set *P* values considered significant at .05 / 9 = .006.^24^

Three authors (M.S.I., D.B.K., and D.P.W.) held group discussions to become familiar with data, identify initial themes, generate initial codes, and create a codebook. These authors independently coded 3 randomly selected transcripts and met to calibrate results. After codebook development, 2 authors (M.S.I. and D.B.K.) coded all the transcripts, and another author (D.P.W.) arbitrated discrepancies. All study authors reviewed final themes to verify accurate representation.

One author (M.S.I.) prepared a textual narrative to summarize findings, while other authors confirmed these narratives. Creswell states that qualitative research’s rigor depends on verification—“the process of checking, confirming, making sure, and being certain and refers to the mechanisms used during the process of research to incrementally contribute to ensuring reliability and validity.”^25^ We verified the data by ensuring methodological coherence, collecting and analyzing the data concurrently to reflexively adjust, interviewing the correct sample population up to thematic saturation, and thinking theoretically, which “required macro-micro perspectives, inching forward without making cognitive leaps, constantly checking and rechecking, and building a solid foundation.”^22^

## Results

We interviewed 47.2% (17 of 36) of all 2023 US medical school women deans and 17.7% (17 of 96) of all men deans (total number, 34). Within our sample, no dean reported a nonbinary or other gender identity. Among participants, 3 (8.8%) self-identified as African American, 2 (5.9%) as Asian, and 29 (85.3%) as White. About two-thirds of deans had practiced a medicine-based specialty or subspecialty (23 [67.6%]). Medical schools of the deans were evenly distributed among 4 US Census regions: Central (9 [26.5%]), Northeast (8 [23.5%), South (10 [29.4%]), and West (7 [20.6%]). The mean (SD) number of students enrolled at these medical schools was 654.0 (236.9). The mean (SD) interview length was 27.6 (12.2) minutes. Demographic information about medical school rankings, deans’ prior leadership roles, deans’ current spheres of responsibility, and deans’ clinical specialty are presented in eTables 1, 2, and 3 in Supplement 1.

There were no statistically significant differences observed between women and men participants with regard to demographics, such as number of years to attain deanship (mean [SD], 2.7 [3.4] vs 3.7 [3.7] years), number of years as a dean (mean [SD], 5.7 [5.2] vs 60 [5.0] years), highest salary during career (mean [SD], $525 769 [$199 936] vs $416 923 [$195 848]), or medical school rankings (mean [SD], 315.5 [394.5] vs 480.5 [448.9]) (Table 1).^23,24^ Eleven participants had held interim dean positions for a median of 12 months (IQR, 11-18 months [range, 7-34 months]). There was considerable variability in the number of years participants held deanship roles (median, 4.0 years [IQR, 2-11 years]; range, 1-18 years).

**Table 1.** Demographic Characteristics of Women and Men Deans

Demographic variable	No. of deans	Mean (SD) value	*P* value^a^	Effect size^b^
No. of years to attain deanship from first application submission				
Women	12	2.7 (3.4)	.42	−0.25
Men	12	3.7 (3.7)		
No. of years as a dean				
Women	17	5.7 (5.2)	.76	−0.08
Men	17	6.0 (5.0)		
No. of positions applied for				
Women	17	3.7 (2.4)	.66	0.11
Men	17	3.2 (3.2)		
No. of unsolicited applications				
Women	17	0.4 (0.7)	.36	−0.23
Men	17	0.8 (1.6)		
No. of positions nominated for				
Women	17	3.4 (2.4)	.18	0.34
Men	17	2.2 (2.0)		
No. of times as a finalist				
Women	17	1.6 (0.8)	.28	−0.30
Men	17	2.2 (1.6)		
Highest salary during career, not necessarily deanship, $				
Women	13	525 769 (199 936)	.22	0.36
Men	13	416 923 (195 848)		
Research rank total of medical school				
Women	17	315.5 (394.5)	.24	−0.29
Men	17	480.5 (448.9)		
Primary care ranking of medical school				
Women	9	71.9 (32.8)	.97	0.01
Men	9	71.3 (29.4)		

^a^ Bonferroni correction for pairwise comparison: *P* = .05/9 = .006.^24^

^b^ Cohen *d* effect size: 0 through 0.1, no effect; 0.2 through 0.4, small effect; 0.5 through 0.7, intermediate effect; and 0.8 or more, large effect.^23^

In their personal lives, more women (n = 5) than men (n = 2) reported taking leadership positions, including a deanship, even when it meant living separate from their partner or spouse. The women considered this sacrifice necessary due to the limited availability of high-level leadership opportunities and also their partners’ or spouses’ established careers. One man lived apart from his partner because she held a similar influential academic leadership position. Women did not report that living apart from their partner or spouse affected their lives. Universally, both women and men reported that unwavering partner or spousal support played a pivotal role in their career advancement and taking on the dean role. None of the participants had caretaking or geographic restrictions that affected their decision to take on the deanship. All participants with children reported that their children were of adult age by the time they became dean.

The qualitative results revealed 3 substantive gender differences in the path to deanship. First, women emphasized that they had to work very hard and be proactive in promoting themselves to attain the decanal position, whereas men often discussed being nurtured for this role. One woman dean stated, “Nobody’s ever taken me under their wing to say, ‘Let me help you get there’” (participant 23), exemplifying that she had to have significant self-determination to attain the deanship, as compared with a man dean who stated, “I certainly knew that I was on the pathway to becoming a dean” (participant 22). Unlike the men, the women suggested that they were rarely recruited for opportunities that would prepare them for a dean’s position. For instance, 1 man pointed out that he “did not apply to be a board member for [X], they asked me to apply” (participant 7). This individual also attributed these opportunities to his mentors*:* “I’ve had phenomenal mentors who see things in me and see positions that I should be considering that I would not otherwise have considered.” Men deans also reported that they were offered stepping-stone leadership positions early in their career, indicating that early career cultivation may have played a role in eventually attaining the deanship. In fact, one male dean stated that he was “early in his 40s, when [a senior mentor] started promoting leadership opportunities for me” (participant 26).

Women deans, unlike men, uniformly expressed both an obligation and a strong desire to participate in leadership development programs to advance their careers. One woman dean stated, “I was in the first ELAM [Executive Leadership in Academic Medicine] class. Many years ago, I saw the ad for the program and went to my dean and said, ‘I want to do this.’ He did not object, so I did it. The ELAM experience was pivotal in preparing me to become a dean” (participant 15). On the other hand, illustrative comments from the men deans suggested the feeling that they did not need professional development, including one who said, “I’m going to be honest, and on many days it shows, but I did not participate in any professional [leadership] development programs” (participant 9), or another man who stated, “I’ve been a leader literally throughout my whole life. I was a serious athlete, and I think I still am in a way. I was always captain of the team, so I never took a leadership course or anything like that” (participant 17).

Finally, women commonly described gender bias emanating from search firms. In fact, all women in this study went through national search processes to attain their positions, and many described facing tokenism based on their gender and/or race and ethnicity. A few women reported that they did not receive complete preparatory information from search firms. For instance, one woman (participant 12) stated, “Once I had a very senior White male tell me how it works…that I would walk in loaded with all the information I could gather. You needed to answer the questions, and that’s a totally different game and one that women don’t necessarily get [or win].” On the other hand, few men participants complained about search firms, and many reported that there was “no search” involved during their deanship appointment, with one man dean stating, “Actually, I didn’t even go through an interview process because [a senior leader] knew me so well” (participant 17). Table 2 provides illustrative quotes of these 3 themes.

**Table 2.** Gendered Differences in the Path to Deanship

Women		Men	
Theme	Representative Quotes	Theme	Representative Quotes
Be proactive to attain deanship	Nobody’s ever taken me under their wing to say, “Let me help you get there.” (Participant 23) Nope, not really. No one coached or mentored me to get to this position, especially early in my career. (Participant 34) I talk about a seat at the table, and that journey to being invited in the room, sitting in the background, and then being invited to sit at the table and then what it takes to gain the courage to speak at the table, and who were the influencers in giving me the courage and confidence to speak, and what the impact…those experiences on my career, I think, were really important influencers and not all women get that. (Participant 19)	Cultivated for deanship	I certainly knew that I was on the pathway to be dean there. (Participant 22) [The] new dean called me to his office and he said, “Would you be willing to be the director of the [x]?” I said, “Well, I mean…I know nothing about [x].” He looked at me and he said, “You seem very good at organizing, who knows; if you do this, you might someday be a dean.” (Participant 28) I think the dean saw something in me, he saw leadership potential. It was also a lot of mentoring from the former deans. Literally daily learning. They would bring me in their inner circle. They involved me with things. I was in on all the high-end conversations, deliberations, and I learned from them. (Participant 3) Well, I was hired actually first to be dean of research, with the idea that within a year or 2 I’d be the overall dean, and that’s what happened. (Participant 17)
Trained to lead	I’m just passionate about career and leadership development. I have done a lot of it. I don’t have them all off the top of my head but ELAM and through the AAMC any kind of communication, conflict management, those kinds of seminars use academic impression in terms of leadership development. I have to say, I am also a huge fan of leadership coaches in general. (Participant 8) I also had just an innate passion to always be a better leader. So, even from the time that I started in academic medicine, I did professional development things related to being a stronger leader, related to improving leadership skills, I constantly read leadership books, I did a leadership development program for our residents and a leadership development program for students. So, I think of myself as a student of leadership in terms of the formal meetings that I did. (Participant 11) I did ELAM and a Healthcare Leadership Institute course with the business school. I also did the Global Institute for Leadership Development. I did Lean process improvement training. I had an executive coach for 2 years as chair, as a new chair. (Participant 1)	Born to lead	I did not participate in any leadership development courses in my career. (Participant 5) I’m going to be honest and say none, which on many days shows, but I did not participate in any of them, at any professional development programs focused on leadership. (Participant 9) I’ve done leadership development courses before I became chair and in my early 2 years as chair, but not for deanship. (Participant 10) I’ve been a leader literally throughout my whole life. I was a serious athlete, and I think I still am in a way. I was always captain of the team, felt very comfortable in leadership roles in terms of leading large research teams. I feel comfortable in becoming a leader in general. I never took a leadership course or anything like that. (Participant 17) I don’t recall a specific…I think my role as a chair for a long time, for almost 20 years, and my role in the dean’s office prepared me for this, but I didn’t do any additional training for that. (Participant 20)
Search firm difficulties	She shared with me there was a distinct possibility that many of these places were checking a box to say that they had spoken with a woman, they had spoken with a person of color, and all of this stuff. It came in a little bit of a surprise to me as like, “Oh, okay. Well, maybe that’s what’s going on.” (Participant 15) I used to joke that I was on…this sounds very cynical. I was on the “women’s list.” (Participant 25) I was told by the search firm that, “You’re in, you’re in, you’re in,” and I got to the visit and it was like weird. Even though the search firm swore up and down they were going to take an external candidate, they took an internal one. (Participant 12) Some of [the search firms] were really peculiar. Like there was one place where I got invited to an interview, and then I got uninvited from the interview, and I thought that was rather weird. (Participant 14)	No search firm difficulties or no search	There was no search. I was just appointed by the board into the dean position. (Participant 20) When you get on this list, it’s like, “You’re on the list.” I’ve been contacted…I’ve been blessed to be contacted about all these jobs and asked to interview for them. (Participant 7) At some point, people started to ask me about being a dean. (Participant 29)

Abbreviations: AAMC, Association of American Medical Colleges; ELAM, Executive Leadership in Academic Medicine.

## Discussion

We found substantive qualitative gender differences associated with career preparation for medical school dean positions, echoing Sandburg’s observation, “Men are promoted based on potential, while women are promoted based on accomplishments.”^26^ Men deans were more commonly asked early in their careers to assume foundational leadership roles that provided stepping stones to the position of dean. This nurturing extended beyond typical sponsorship and involved established leaders preparing and shaping men deans for specific roles through continuous development, mentorship, training, sponsorship, and strategic career moves.^27^ In some instances, men were fast tracked into leadership positions within a few years of completing subspecialty training.

Conversely, women deans reported that their career paths were not mapped out by senior leaders and especially not as early in their careers. This notion of not receiving similar cultivation of their leadership potential is supported throughout the literature. Studies show that women are less likely than men to be asked to serve in leadership roles, such as department chairs or division directors.^28^ For instance, among emergency medicine department chairs, men commonly stated that sponsorship from senior leaders affected their career advancement, whereas women chairs “advanced through their own hard work and effort.”^29^ Women are also infrequently encouraged by superiors to run for institutional elected positions that would contribute to professional advancement.^30^ Simultaneously, women might not be seen as effective leaders by potential sponsors for myriad reasons, including role congruity.^31^ As stated in the study by Hastie et al, “For women in academic medicine, positions of leadership are achieved, despite the presence of challenges, not because of their absence.”^32^

Because the women deans were more likely not to have had their leadership cultivated or been given early access to pivotal sponsors, it is unsurprising that they felt more obligated than men to participate in career development programs to level the playing field. Many of the women deans participated in programs such as the AAMC Mid-Career Women Faculty Professional Development or the Hedwig van Amerigen ELAM programs.^33,34^ Participation empowers individuals with strategies to navigate the often political academic medicine workplace environment and leverage the resources needed to counter the effects of gender bias in speaker invitations, authorships, publication rates, federal grant success, and other factors that build academic credibility.^35,36,37^ Women who participated in such programs were as likely as men and more likely than nonparticipant women to be promoted to full professor within 10 years.^38,39^ Leadership development program participation also expanded participants’ networks and increased visibility, both within and external to their institutions. For women, in particular, participation in leadership development programs led to high-level leadership opportunities and placed them on search firm lists.^39,40,41,42^ Among all current women deans, 63% were graduates of the ELAM program.^34^ Nevertheless, because extramural leadership development programs require the approval and financial support of the home institution, they cannot be a replacement for consistent, deliberate, intramural career development for guiding individuals through the ranks.

Additional hurdles faced by women deans in our study were inherent biases during searches for deanships, including the feeling they were just token candidates. Studies have shown that executive search consultants exclude women candidates, particularly women from racial and ethnic minority groups and those with intersectional identities, at each step of the search process, from identifying and profiling, to shortlisting, and presenting candidates to institutions.^43,44^ On the personal front, more women than men had to make the decision to live separately from their partner, spouse, or family when they attained the deanship. In addition, women deans were more likely to be part of dual-career households and had less flexibility because of their partner’s or spouse’s careers, thus accepting “commuter relationships.”^45^

Our results suggest that efforts to cultivate leadership potential should be specifically targeted toward aspiring midcareer women leaders.^16,46^ As compared with early-career women, midcareer women risk becoming invisible because of waning professional attention and support.^47^ One possible explanation is that as midcareer women gain experience and accomplishments, they become more threatening and are subsequently dismissed based on a variety of gender stereotypes.^48^ Another reason is that women are more likely to be “voluntold” to take on nonpromotable housekeeping activities, including work in education; mentoring; and diversity, equity, and inclusion, with an overemphasis on service to others that can derail career advancement.^5,6,7,49^ These areas of medicine are historically not as respected as research, leading to further micro- and macro-inequities.^50,51,52,53,54^

The consequences of women constantly needing to be proactive and solely responsible for advancing their careers includes burnout, decreased job satisfaction and sense of belonging, transitioning to part time, poor work-life integration, and attrition.^53,55,56^ Research suggests that women are more likely than men to leave academic medicine.^57,58^ For instance, a recent study^59^ found that women with a lower sense of belonging were more likely to leave their institution in the near future—a retention concern linked to promotion and career advancement opportunities.^58,59,60^

In response, institutions, funding agencies, and professional societies should invest in the development of all faculty, paying particular attention to midcareer women. For example, in the United Kingdom, the Athena Scientific Women’s Academic Network (SWAN) Charter holds institutions accountable for efforts in advancing women in science, technology, engineering, math, and medicine, and institutional support from some funding bodies is tied to the level of Athena SWAN awards held.^61^ Medical schools might consider strategies such as implementing term limits to allow for women to successfully attain experiences that position them well for deanships, while also promoting diversity, equity, and inclusion within their school.^2,62,63^ The NIH introduced a 12-year term limit for intramural laboratory and branch chiefs to diversify leadership by opening up positions for women and members of racial and ethnic minority groups, which is another positive step.^64^ Finally, to mitigate some of the barriers women face, institutions should formally sponsor women for stepping-stone leadership positions and incorporate metrics such as the gender proportionality principle (which advises that a given level in an organization should reflect the gender composition immediately below it).^65,66^

### Limitations

This study had a few limitations. Although we had participation success with an often hard to reach population, we were not able to interview every US medical school dean in 2023. Therefore, the results may not be generalizable, although we recruited nearly half of all women deans. Although we strived for equitable representation, based on our sampling strategy, we were also not able to interview every one of the few deans belonging to a racial and ethnic minority group. We also did not interview candidates who never attained a deanship, who very well may have experienced differences in their treatment or qualifications associated with gender. Finally, our study was underpowered to perform inferential statistical tests, including evaluating whether there were differences between women and men in their spheres of responsibility in dean roles. Subsequently, our quantitative analyses should be interpreted only within the context of confirming the findings of others’ comparisons of gender associated with deans’ demographics.

## Conclusions

In this qualitative study, we found meaningful differences in the experiences of women and men on their paths to becoming medical school deans. In particular, the stories from our study show that women had to be more proactive and work harder than men to attain the deanship. Women attributed their need for this extraordinary effort to a lack of anyone cultivating their leadership potential early in their careers and lack of access to career development networks. This gender bias may have negative consequences for rising women leaders, including burnout and attrition, thus potentially affecting the makeup of future generations of medical school deans. Our study highlights the need for equivalent leadership development efforts for both women and men.

## Notes

Supplement 1. **eAppendix.** Telephone Interview Guide **eTable 1.** Institutional Rankings of the Medical Schools Represented by the Deans Broken Down by Gender **eTable 2.** Deans’ Prior Leadership Roles and Current Spheres of Responsibility **eTable 3.** Number and Percentage of Deans by Clinical Specialty and Gender

Supplement 2.
Data Sharing Statement
