# Error in Author Affiliations

**DOI:** 10.1001/jamanetworkopen.2024.31056

**Published:** 2024-08-05

In the Original Investigation titled “Gender Differences in the Path to Medical School Deanship,”^1^ published July 5, 2024, the institutional affiliation for Dr Gottlieb was incorrect. Dr Gottlieb was affiliated with the Departments of Obstetrics and Gynecology and Internal Medicine at Keck School of Medicine, University of Southern California, Los Angeles. This article has been corrected.^1^
