# Supplementary material for: Gender Differences in the Path to Medical School Deanship
Source: JAMA Netw Open. 2024 Jul 5;7(7):e2420570. doi: 10.1001/jamanetworkopen.2024.20570 (PMC11227086; doi:10.1001/jamanetworkopen.2024.20570)
Supplement: Supplement 1. — eAppendix. Telephone Interview Guide eTable 1. Institutional Rankings of the Medical Schools Represented by the Deans Broken Down by Gender eTable 2. Deans’ Prior Leadership Roles and Current Spheres of Responsibility eTable 3. Number and Percentage of Deans by Clinical Specialty and Gender [file jamanetwopen-e2420570-s001.pdf]

Supplementary Online Content

Iyer MS, Bradford C, Gottlieb A, et al. Gender differences in the path to medical school deanship. *JAMA Netw Open.* 2024;7(7):e2420570. doi:10.1001/jamanetworkopen.2024.20570

eAppendix. Telephone Interview Guide
eTable 1. Institutional Rankings of the Medical Schools Represented by the Deans Broken Down by Gender
eTable 2. Deans' Prior Leadership Roles and Current Spheres of Responsibility
eTable 3. Number and Percentage of Deans by Clinical Specialty and Gender

This supplementary material has been provided by the authors to give readers additional information about their work.

© 2024 Iyer MS et al. *JAMA Network Open.*

eAppendix. Telephone Interview Guide

Path to Deanship Research Project

Introduction:

Thank you for agreeing to participate in this interview. Today, we are going to talk about your path to becoming a Dean. During this interview, we would like to focus on your individual experiences and stories, specifically as someone who has sought and attained leadership positions in medicine. We want to understand your experiences and use your insights to help guide others seeking to develop productive careers like yours.

As a reminder, this interview will be recorded. By participating in this interview, you are providing verbal consent. What we discuss today is confidential and this information will not be shared with anyone outside of the study team. Your name/identity will not be attached to any information that you provide. Should names of other individuals or organizations accidentally be disclosed, they will be removed in the final data analysis. In addition, you have the right to skip any questions or stop the interview. May I ask you to acknowledge that I have explained the interview process to you and that you consent to proceeding with the audio recorded interview? Do you have any questions before we start?

Path to Deanship Interview Questions

1. When and why did you decide to become the Dean?

2. What are your responsibilities in your current Dean role?
    1. Medical School only?
    2. Medical School and Faculty Practice Plan?
    3. Medical School and at least one other health professions school?
    4. Medical School and Hospital or Health System (EVP/CEO)?
    5. Combination of the above?

    Follow Up: Is this the same as what you were initially hired to do?

3. Tell me if you held any of the following leadership positions before becoming the Dean: Vice Dean, Associate Dean, Department Chair, center or institute director, or other similar senior administrative position in academic medicine. *Please note administrative area(s) for decanal positions and department chair roles.*

    Follow Up: What was your sphere of responsibilities in these prior roles?
    1. Medical School only?
    2. Medical School and Faculty Practice Plan?
    3. Medical School and at least one other health professions school?
    4. Medical School and Hospital or Health System (EVP/CEO)?
    5. Combination of the above?

4. Have you ever held an Interim Dean role? If so…
    a. How long was your tenure as Interim?
    b. What happened next? (e.g., did you transition to permanent Dean at the same institution or permanent Dean somewhere else)?
    c. If you did not transition to permanent Dean at the same institution, why not (specifically, what was the context – was someone else selected or did you decline the offer?)

5. Can you describe any professional development conferences, programs, or activities in which you participated and believed played a role in your leadership career?

6. Is there anything else that you thought really prepared you to take on the role as Dean?

© 2024 Iyer MS et al. *JAMA Network Open.*

We want to understand more about personal factors that may have affected your path to the Dean role.

1. Did you have any caregiving responsibilities (e.g., child care, elder care, or caring for ill partner, family member, or friend) that may have influenced your considerations in becoming Dean?

2. How would you describe your geographic mobility at the time of your Dean appointment? (ie: family, academic considerations)?

3. Are there any other personal characteristics you think influenced your path to becoming a dean?

We would like to ask some briefer background questions before we conclude this interview.

1. How many years have you served as the Dean at a single medical school?

2. Have you served as the Dean at another medical school? And if so, how long?

3. How many Dean positions did you apply for prior to accepting your current Deanship?
    a. How many of your applications were ***nominated or solicited*** *(ie: through a search firm, word of mouth, professional network connections)?*
    b. How many of your applications were ***unsolicited*** *(ie: you threw your name in the hat)*?

4. How many years did it take to attain your first Dean position from the first application you submitted?

5. For how many of the positions to which you applied were you invited to interview?
    a. How many times did you get to an initial "airport" interview or search committee interview, but not beyond?
    b. How many times did you get to final interviews at the president/provost level and were not selected?

6. What is the most education debt you had prior to becoming the Dean?

7. What was the highest level of compensation per year you received prior to becoming the Dean?

8. How would you describe your gender?

9. What is your ethnicity?

10. How would you describe your race?

Closing

Now that we've concluded the interview, do you have any other comments or questions for me at this time?
Thank you again for sharing your story with me today. It was really a pleasure to speak with you.

© 2024 Iyer MS et al. *JAMA Network Open.*

eTable 1. Institutional Rankings of the Medical Schools Represented by the Deans Broken Down by Gender

		N	Mean (SD)	Median	IQR
**Blue Ridge Ranking[1]**	Men	16	78.4 (38.1)	80.5	63
	Women	15	59.7 (37.3)	48	60
	Total	31[a]	69.4 (38.3)	72.0	57.0
**USNWR-Research[2]**	Men	10	53.2 (29.6)	51.5	66
	Women	14	57.9 (36.1)	66	57
	Total	24[b]	56.0 (32.9)	58.5	58.0
**Composite Research Rankings[3]**	Men	10	117.5 (52.0)	116	107
	Women	13	105.2 (62.2)	91	89
	Total	23[c]	110.6 (57.1)	105.0	85.0
**USNWR-Primary Care[2]**	Men	17	68.2 (29.4)	62.5	55
	Women	17	74.4 (34.8)	71	68
	Total	23[c]	71.7 (32.0)	64.0	61.0

Please note that all unranked institutions were eliminated from these data: [a]**N=3 unranked;** [b]**N= 10 u**nranked; [c]N= 11 unranked

1. The Blue Ridge Institute for Medical Research (BRIMR). Retrieved from https://brimr.org/. Accessed November 11, 2023.
2. U.S News and World Report 2023-2024 Best Medical Schools-US News. Retrieved from https://www.usnews.com/best-graduate-schools/top-medical-schools/primary-care-rankings. Accessed November 11, 2023.
3. Composite Research Rankings were created by adding Blue Ridge and USNWR-Research Rankings.

eTable 2. Deans' Prior Leadership Roles and Current Spheres of Responsibility
**An individual may have more than one prior role/sphere of responsibility*

	**Women Deans (n)**	**Men Deans (n)**
**Role**		
Dean at Another Institution	1	4
Interim Dean	5	6
Regional Campus Dean	1	1
Department Chair	10	8
Division Chief	4	5
Vice Chair	2	0
Medical Staff Leadership	1	3
Center/Institute Director	4	0
Executive/Vice Dean	3	5
Associate/Assistant Dean	6	5
Fellowship/Residency/Postgraduate Program Director	3	4
**Sphere of Responsibility**		
Medical School Only	5	2
Health Professional Schools	3	7
Graduate Schools	1	2
Faculty Practice Plan	3	8
Laboratory/Center	0	1
Service Line	1	0
C-Suite (ie Chief Medical Officer)	2	1
Health System (ie: President, VP Health Affairs)	6	11
Provost/Associate Provost/Chancellor	4	4

© 2024 Iyer MS et al. *JAMA Network Open.*

eTable 3. Number and Percentage[a] of Deans by Clinical Specialty and Gender

Specialty	Women Deans	Men Deans	TOTAL
Medical (anesthesiology, emergency medicine, family medicine, internal medicine, neurology, pediatrics, radiology, psychiatry)	10 (58.8)	13 (76.5)	23 (67.6)
Surgical (general, otolaryngology, Obstetrics/gynecology, plastics, vascular)	7 (41.2)	4 (23.5)	11 (32.4)
TOTAL	17 (50)	17 (50)	34 (100)

[a]Column Percentages

© 2024 Iyer MS et al. *JAMA Network Open.*
