# Supplementary material for: Gender Differences in the Path to Medical School Deanship
Source: JAMA Netw Open. 2024 Jul 5;7(7):e2420570. doi: 10.1001/jamanetworkopen.2024.20570 (PMC11227086; doi:10.1001/jamanetworkopen.2024.20570)
Supplement: Supplement 2. — Data Sharing Statement [file jamanetwopen-e2420570-s002.pdf]

# Data Sharing Statement

Iyer. Gender Differences in the Path to Medical School Deanship. *JAMA Netw Open*. Published July 05, 2024. doi:10.1001/jamanetworkopen.2024.20570

**Data**
**Data available:** No

**Additional Information**
**Explanation for why data not available:** We cannot share the data because the data are easily re-identifiable and would jeopardize subject confidentiality.
